# Sediment potentially controls in-lake phosphorus cycling and harmful cyanobacteria in shallow, eutrophic Utah Lake

**DOI:** 10.1371/journal.pone.0212238

**Published:** 2019-02-14

**Authors:** Matthew C. Randall, Gregory T. Carling, Dylan B. Dastrup, Theron Miller, Stephen T. Nelson, Kevin A. Rey, Neil C. Hansen, Barry R. Bickmore, Zachary T. Aanderud

**Affiliations:** 1 Department of Geological Sciences, Brigham Young University, Provo, UT, United States of America; 2 Department of Plant and Wildlife Sciences, Brigham Young University, Provo, UT, United States of America; 3 Wasatch Front Water Quality Council, Salt Lake City, UT, United States of America; Chinese Academy of Sciences, CHINA

## Abstract

Lakes worldwide are impacted by eutrophication and harmful algal or cyanobacteria blooms (HABs) due to excessive nutrients, including legacy P released from sediments in shallow lakes. Utah Lake (northern Utah, USA) is a shallow lake with urban development primarily on the east side of the watershed, providing an opportunity to evaluate HABs in relation to a gradient of legacy sediment P. In this study, we investigated sediment composition and P concentrations in sediment, pore water, and the water column in relation to blooms of harmful cyanobacteria species. Sediments on the east side of the lake had P concentrations up to 1710 mg/kg, corresponding to elevated P concentrations in pore water (up to 10.8 mg/L) and overlying water column (up to 1.7 mg/L). Sediment P concentrations were positively correlated with Fe_2_O_3_, CaO, and organic matter abundance, and inversely correlated with SiO_2_, demonstrating the importance of sediment composition for P sorption and mineral precipitation. Although the sediment contained <3% Fe_2_O_3_ by weight, approximately half of the sediment P was associated with redox-sensitive Fe oxide/hydroxide minerals that could be released to the water column under reducing conditions. Cyanobacteria cell counts indicate that blooms of *Aphanizomenon flos-aquae* and *Dolichospermum flosaquae* species tend to occur on the east side of Utah Lake, corresponding to areas with elevated P concentrations in the sediment, pore water, and water column. Our findings suggest that shallow lake eutrophication may be a function of P in legacy sediments that contribute to observed HABs in specific locations of shallow lakes.

## Introduction

Harmful algal or cyanobacteria blooms (HABs) remain a substantial water quality concern as reoccurring blooms impact human health, cause a decline in lake recreation value, and create ecological problems, especially in shallow lakes that are prone to water level fluctuations and eutrophication. HABs are triggered by increased levels of total lake phosphorus (P) and nitrogen (N) from anthropogenic nutrient pollution [1]. HABs are especially problematic in unstratified shallow lakes because of strong interactions between the water and land, atmosphere, and sediment [2–4]. However, many HAB dynamics remain elusive, such as the amount of total lake P necessary to produce blooms in response to external P loading or internal P release from legacy sediments [5]. Internal P fluxes from sediments to the overlying water column often result in time lags for restoration of shallow lakes after reduction in external nutrient loads [6–10].

Shallow lakes are prone to P release given high surface area to volume ratio, making sediment-water interactions a key process in dissolved P exchange [4, 8]. Sediments in shallow lakes act as a net P sink [11] but may serve as a temporary P source depending on the physicochemical properties of the sediments and overlying water [12]. P release is regulated by the interactions among dissolved oxygen, pH, temperature, microbial activity, and pore water P concentrations [11, 13]. Reducing conditions in sediment drive reductive dissolution of Fe (oxy)hydroxide minerals, releasing Fe oxide-bound P from the sediment [14]. Loosely bound P in sediments or dissolved P in pore water become available after resuspension by carp bioturbation or wind [15, 16]. These chemical and physical processes underscore the need to understand P speciation in sediments and interactions with water chemistry.

The interconnectedness of P in sediments and the water column may influence HABs. The microbial species responsible for the deleterious effects of HABs are due to cyanobacteria since cyanobacteria alone produce cyanotoxins, which are the primary water quality concern. To bloom, certain cyanobacterial species may exploit the relatively cooler temperatures in spring and/or shady versus sunny conditions [17]; others may generate their own nitrogen as N-fixers and outcompete species relying on inorganic N [18]; and still others may rely on different forms of P (i.e., soluble reactive P, organic P, mineral occluded P) [19]. Often it is a combination of conditions that cause certain cyanobacteria to bloom, but these conditions are consistently linked to some form of P. For example, modeling of distribution of six cyanobacterial species in five shallow eutrophic lakes over two years, the abundance of five cyanobacteria (two *Planktothrix*, two *Aphanizomenon*, and an *Anabaena* species) were linked to total P, and two species (*Aphinizomenon* and *Anabaena* species) to soluble reactive P in the water column [17]. The exact amount of total lake P to elicit blooms varies substantially due to the internal storage and cycling of P in sediments. Thus, with the potential for shallow lakes to internally cycle P and the sensitivity of cyanobacteria to different forms of P, the interconnectedness of P in sediments and water column may dictate blooms.

In this study, we investigated P concentrations and speciation with potential links to HAB production in Utah Lake, a shallow, eutrophic, freshwater lake experiencing lake closures in recent years due to HABs. Utah Lake is the third largest freshwater lake in the western U.S. with an area of 375 km^2^ but an average depth of only 3 m (maximum depth of 6 m) under normal lake water levels. Nearly all the urban development is located on the east side of the watershed, providing an opportunity to evaluate HABs in relation to a gradient of P concentrations in the water column and legacy sediments between the east and west sides of the lake. To understand the linkages between P and HABs, our study was undertaken to: (1) evaluate P concentrations in sediment, pore water, and the water column; (2) characterize sediment composition, P speciation, and potential for P release; and (3) compare P spatial variability with counts of three common cyanobacterial species creating HABs in Utah Lake. Further, to investigate the impacts of urban nutrient inputs, special attention was given to sediments near the wastewater treatment plant (WWTP) outfalls occurring on the east side of Utah Lake.

## Materials and methods

### Study area

**Fig 1:**
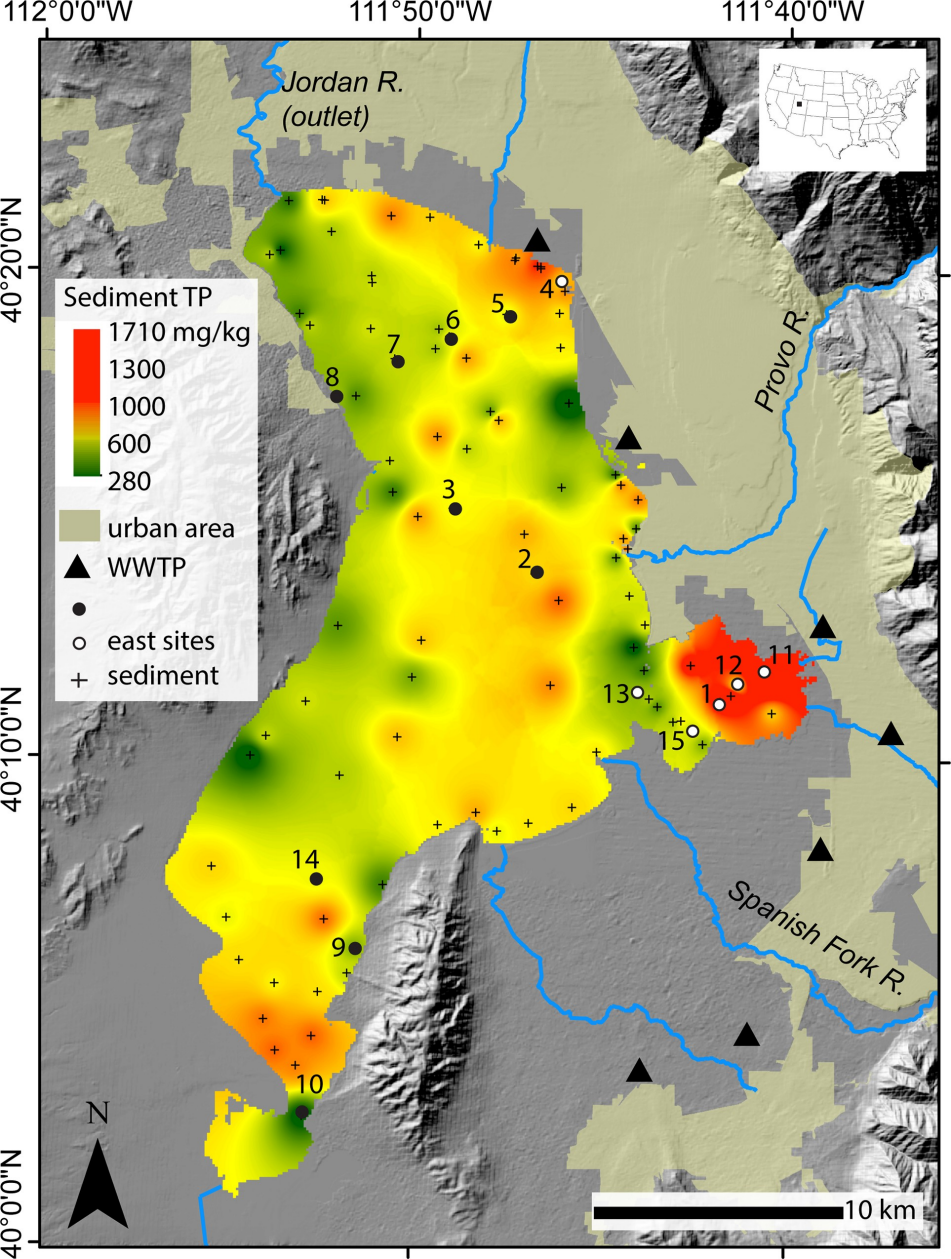
Location map of Utah Lake. Map shows interpolated 2015–2016 total phosphorus (TP) concentrations in Utah Lake sediments, the Wasatch Front urban area, wastewater treatment plants (WWTPs), tributaries, and the Jordan River outlet. Sample locations identified with circles and number label were sampled for surface water, pore water, and sediment (black circle = west and white circle = east) while sample locations identified with a cross were sampled for sediment only by Abu-Hmeidan et al. [21]. The urban area is defined according to the 2010 US census.

Utah Lake (Fig 1), a remnant of Pleistocene Lake Bonneville, is located in rapidly urbanizing Utah Valley, with a population >500,000 on the east side of the lake that is expected to double by 2050. The lake is popular for recreation but was closed during portions of 2016 and 2017 due to the presence of HABs. As a basin bottom lake in a rapidly urbanizing area, the lake receives nutrients from agricultural runoff, wastewater effluent, natural P in the local geology, and atmospheric deposition [20–22]. Seven WWTPs discharge into the east side of Utah Lake and waste streams are only expected to grow with increasing population. In particular, Provo Bay (Fig 1) receives effluent from three WWTPs. The majority of surface water inputs occur on the east side of the lake, including the Provo and Spanish Fork Rivers (Fig 1). Sedimentation rates average about 1.4 mm/yr—primarily from precipitation of carbonate minerals from the alkaline lake water [23]—potentially providing an important control on P concentrations in the water column by co-precipitation.

To combat eutrophication and HABs, the Utah Division of Water Quality has proposed a new rule to limit P in WWTP effluent to 1 mg/L by 2020 [24] and ultimately to 0.1 mg/L, at a capital cost ranging from $24 million to over $1 billion statewide depending on the level of treatment [25]. Given the legacy loading of P to Utah Lake, it is unclear whether this increased treatment would lead to immediate or long-term improvements in water quality.

### Water and sediment sampling

To evaluate the potential for P cycling in Utah Lake, we measured P concentrations in three interacting lake compartments: sediment, pore water, and the water column. A total of 26 co-located samples of all three compartments were collected from 15 sites across Utah Lake during October 2015 (“A”), May/June 2016 (“B”), August 2016 (“C”), and November 2016 (“D”), with an emphasis on sediment near the effluent of WWTPs on the east side of the lake (Fig 1). Sample sites 1, 4, 11–13, and 15 (“east”) were located near WWTP inputs and sample sites 2–3, 5–10, and 14 (“west”) were located in the main body of the lake. Not all sites were visited during each sampling event, resulting in thirteen samples from both the east and west side of the lake. While the east versus west groupings are somewhat arbitrary, we found that it was the most useful way to separate the sites based on proximity to urban nutrient sources on the east side of Utah Lake. Lake sediments were collected using a stainless-steel Ekman dredge to isolate the top 5 cm of sediment, and pore water was extracted by centrifugation. Water column samples were taken from the middle of the water column using a peristaltic pump with FEP tubing. No specific permission was required for the field work because Utah Lake is located on public state land and the sampling did not include endangered or protected species.

### Sediment mineralogy and chemistry

To characterize sediment composition, we measured TP, organic matter, carbonate abundance, mineralogy and trace/major element chemistry in all samples (*n* = 26). TP was analyzed by total digestion using USEPA Method 3052. Briefly, 8 mL concentrated HNO_3_ and 2 mL concentrated H_2_O_2_ were added to 0.1 g of sediment and microwave digested at 180°C for 15 min for TP analysis by Thermo Scientific iCAP 7400 ICP-OES. Organic matter and carbonate abundance were determined by mass loss on ignition (LOI) after combustion in a muffle furnace at 550°C and 1000°C, respectively, for 4h [26]. Mineralogy was determined using a Rigaku MiniFlex 600 x-ray diffraction (XRD) instrument. XRD patterns were evaluated using Rietveld methods in the Rigaku PDXL2 software using crystallographic information files obtained from the American Mineralogist Crystal Structure Database. Minerals with <3% abundance were excluded, and mineral abundances were normalized to 100% after adding organic matter from LOI 550°C measurements. An example XRD pattern is provided in the Supporting Information (S1 Fig). Major and trace element concentrations in sediment were analyzed by x-ray fluorescence (XRF) with a Rigaku Primus wavelength dispersive instrument on samples that were powdered, compacted into pressed pellets, and melted into glass disks. The XRF provided major element oxide weight percentages (SiO_2_, TiO_2_, Al_2_O_3_, Fe_2_O_3_, MnO, MgO, CaO, Na_2_O, K_2_O, and P_2_O_5_) and trace elements concentrations (Ba, Ce, Cl, Cr, Cu, F, Ga, La, Nb, Nd, Ni, Pb, Rb, S, Sc, Sm, Sr, Th, U, V, Y, Zn, and Zr). Oxide weight percentages were recalculated after adding carbonate abundance from LOI 1000°C measurements. Scanning electron microscope (SEM) images were taken for sample 1-A (collected from the middle of Provo Bay) to provide an in-situ and non-invasive view of elemental composition at the sediment grain surfaces. Scanning images and dot maps of Ca, Fe, and P were created using energy dispersive spectroscopy capabilities of the XL30 FEI environmental SEM. Raw TP concentrations and XRF/XRD data are provided in the Supporting Information (S1 and S2 Tables, respectively).

### Sequential extraction of sediment samples

Sediment samples from May–June 2016 (*n* = 10) were subjected to a sequential extraction procedure to investigate P speciation in lake sediments. Sediment P species were determined using a sequential extraction scheme for calcite rich lakes from Hupfer et al. [27], modified after Psenner et al. [28], on 2.5 g of wet sediment. Extraction steps included: 1) 1 M NH_4_Cl (deoxygenated–N_2_ purged) shaken for 0.5h to extract P in pore water and loosely adsorbed to surfaces; 2) 0.11 M BD (bicarbonate/dithionite-buffered to a pH 7 using NaHCO_3_) shaken for 1 h to remove redox-sensitive P mainly bound to oxidized Fe and Mn compounds; 3) 1M NaOH shaken for 16 h to remove P exchangeable against OH^-^ ions and P in organic matter; 4) 0.5 HCl shaken for 16 h to remove P in calcium phosphate minerals and acid-soluble organic P; and 5) 1 M boiling HCl for 0.25 h after a 550°C ignition for 2 h to evaluate the refractory organic P and nonextractable mineral P. The sediment was rinsed with 1 M NH_4_Cl between each extraction steps. After each extraction step, the supernatant (including the 1 M NH_4_Cl rinse) was filtered through a 0.45-μm nylon filter and analyzed for TP by ICP-OES. Raw data from the sequential extraction experiments are provided in the Supporting Information (S3 Table).

### Pore water and water column chemistry

To evaluate the relatively mobile form of P in Utah Lake, we measured total dissolved P (TDP; defined as the P fraction passing through a 0.45 μm filter) and trace/major element chemistry in pore water and water column samples. Filtered water samples were analyzed for TDP and other trace/major elements (As, Ca, Ba, Fe, K, Mg, Mn, Mo, Na, Si, Sr, and V) by ICP-OES. Major anions (F^-^, Cl^-^, NO₃^-^, HPO₄^2-^, SO₄^2-^) were analyzed using a Dionex ICS-90 ion chromatograph. Alkalinity (assumed to be HCO_3_^-^) was measured on water column samples by titration. Major ion charge balances were within an acceptable charge balance error of ±5% for all water column samples (S4 Table). Raw water column and pore water chemistry data, including major ion and trace element concentrations, are provided in the Supporting Information (S4 and S5 Tables, respectively).

### HAB species abundance

To identify the potential influence of P on HABs, we evaluated the abundance of total cyanobacteria and three dominant cyanobacteria species in relation to P concentrations across Utah Lake. Total cyanobacteria and specific species were counted via semi-automated imaging flow cytometry (Phycotech Inc.). All samples were collected during the summers of 2016 and 2017 from the upper 0.5 m of the water column and preserved with Lugols solution prior to shipment for counting. Cell counts were collected as part of the HAB monitoring project carried out by the Utah Division of Water Quality and values are publicly available (https://deq.utah.gov/Divisions/dwq/health-advisory/harmful-algal-blooms/bloom-events/index.htm). Some of the highest cell counts were measured at the marinas (Lindon, Provo, and Lincoln Beach; S2 Fig) but they were excluded from our analyses because we lacked P data at these sites. The marinas are small, semi-closed systems that may not represent processes occurring in the main body of the lake or Provo Bay.

### Statistics

To investigate relationships between TP concentrations and sediment composition (i.e., organic matter, CaO, SiO_2_, Fe_2_O_3_), we conducted linear regression analyses using the *lm* function in R (http://www.R-project.org). Further, we tested for differences in TDP concentrations in the water column and pore water between the west and east side of Utah Lake with t-tests in R.

## Results

### Sediment total phosphorus highest on east side of Utah Lake

Sediment TP concentrations in Utah Lake ranged from 280 to 1710 mg/kg, with higher concentrations on the east side of the lake relative to the west side (Fig 1). To create the map shown in Fig 1, we combined our results with those of Abu-Hmeidan et al. [21], who collected 84 sediment samples over the same time period and analyzed TP in the same laboratory as our samples. Elevated TP concentrations (>900 mg/kg) were found in sediment near WWTP effluent, particularly in Provo Bay. Total P concentrations in sediment ranged from 300–900 mg/kg throughout much of the lake, even far from WWTP inputs, representing a moderate to high background in the lake sediments. Notably, parts of the east shore near the inlet of Provo River and Spanish Fork River had low sediment TP concentrations.

### Relationship between sediment composition and TP concentrations

**Fig 2:**
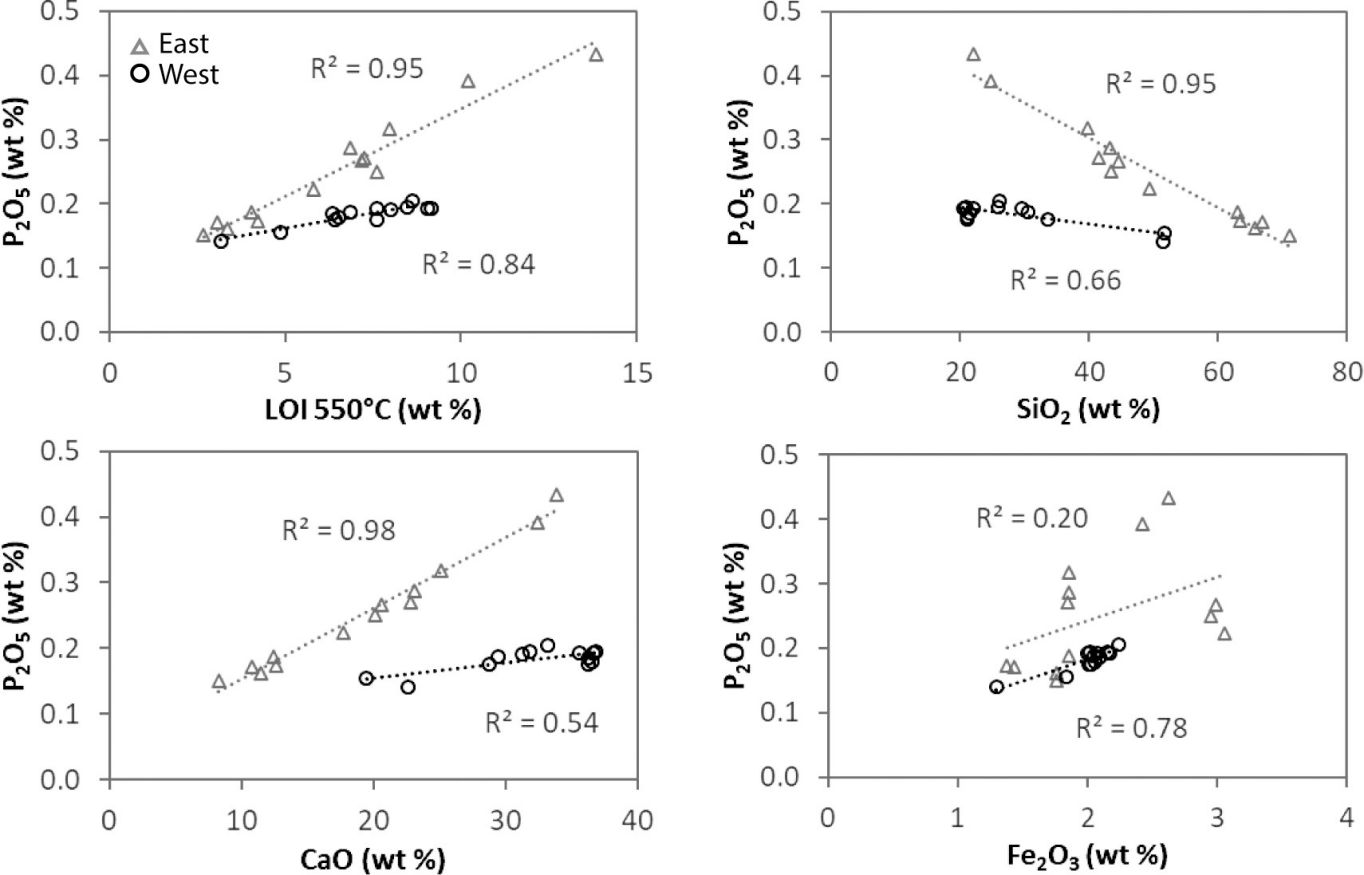
P_2_O_5_ abundance versus LOI 550°C, CaO, and SiO_2_. LOI 550°C is a measure of organic matter concentration. West samples (*n* = 13) were collected from sites 2–3, 5–10, and 14 and east samples (*n* = 13) were collected from sites 1, 4, 11–13, and 15.

In addition to sample location, TP concentrations were partially controlled by sediment composition. Specifically, P_2_O_5_ concentrations were positively correlated with organic matter (measured as LOI 550°C) and CaO abundance, and inversely correlated with SiO_2_ (Fig 2). The relationships were especially strong in the east side samples, with R^2^ values ≥0.95 (F_1,11_ > 200, p < 0.001) for P_2_O_5_ versus LOI 550°C, CaO, and SiO_2_. In contrast, the relationships between P_2_O_5_ and organic matter, CaO, and SiO_2_ were significant but weaker for the west side samples (R^2^ values ranging from 0.54 to 0.84, F_1,11_ < 60, p < 0.005). P_2_O_5_ was also positively correlated with Fe_2_O_3_ abundance, although this relationship was not significant for the east side samples.

**Fig 3:**
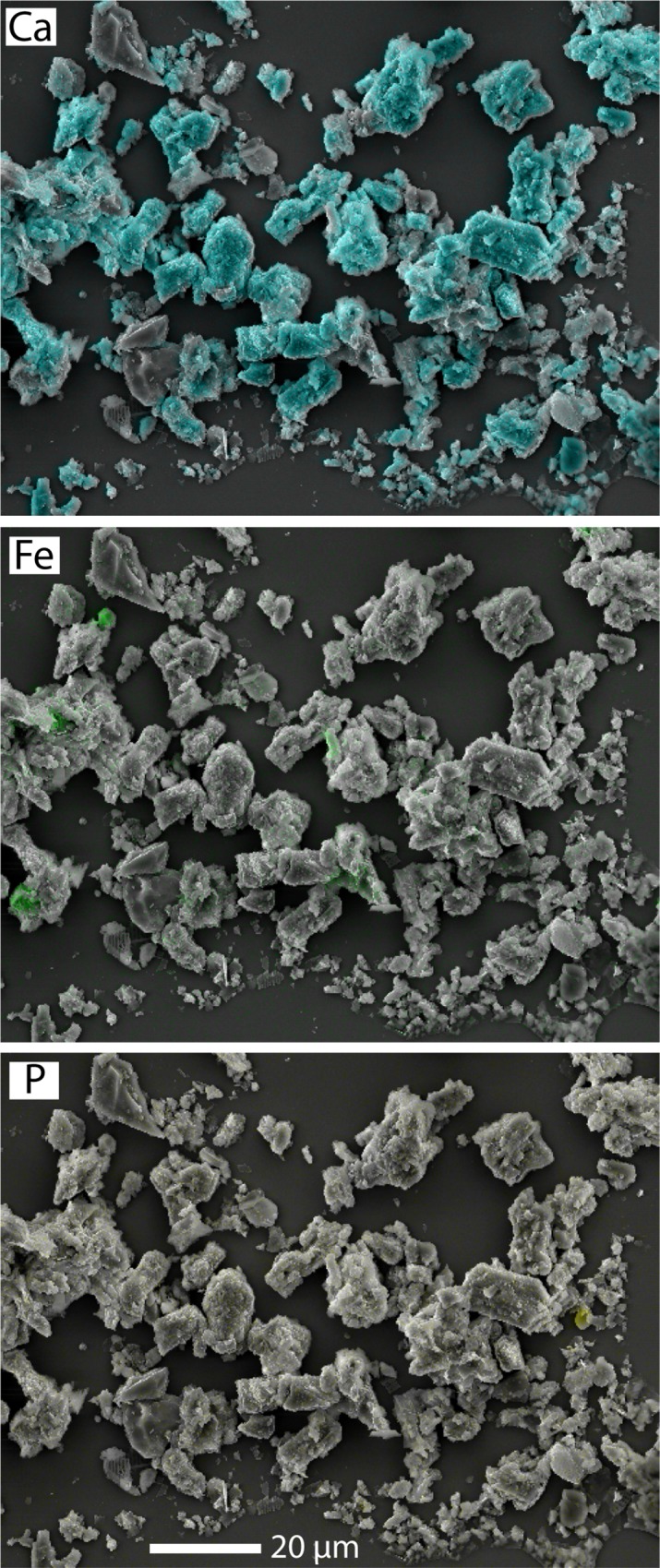
Backscattered SEM image of Utah Lake sediment. The sample is overlain with energy dispersive spectroscopy dot maps of Ca (blue), Fe (green), and P (yellow). Image was taken for sediment sample 1-A collected from the middle of Provo Bay in October 2015.

The XRD analysis shows that sediments are primarily composed of calcite, dolomite, and quartz, with small amounts of organic matter (S2 Table). Calcite mass ranged from 12.7–75.1% (45.9% ± 4.1%; mean ± standard error), dolomite from 2.0–29.8% (7.8% ± 1.3%), and quartz from 10.3–77.0% (39.5% ± 4.9%). Organic matter (from LOI 550°C measurements) ranged from 2.7–13.8% (6.7% ± 0.6%). Peaks for clay minerals (illite, smectite, and kaolinite) and feldspars were small to absent, indicating low abundance, so they were neglected from the analysis. With the exception of clays and feldspars, our sediment mineralogy results are similar to values reported by Hogsett [29]. Notably, there was no clear evidence for aragonite in any of the samples. Likewise, Fe oxy/hydroxide minerals were in low enough abundance that they were not observed by XRD, which is not unexpected given that Fe_2_O_3_ comprised only ~1–3% of sediment mass based on the XRF data. Although Fe concentrations were low, the presence of Fe was confirmed by the SEM images, which showed the distribution of elemental Ca, Fe, and P in lake sediment (Fig 3).

### Phosphorus speciation in lake sediments

**Fig 4:**
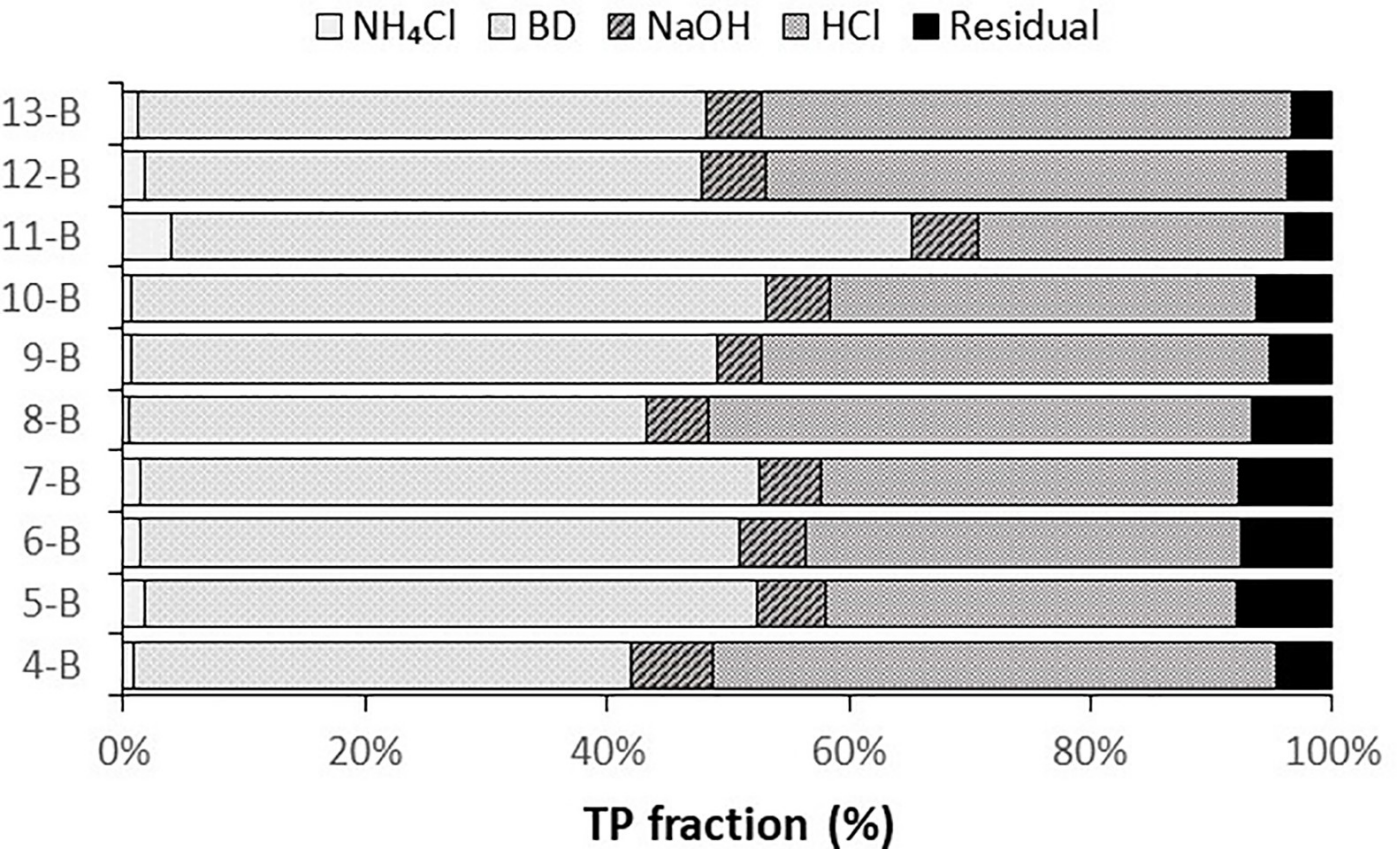
Sediment sequential extraction results. Extraction results for 10 sediment samples collected during May—June 2016. Results are expressed as % total phosphorus NH_4_Cl = P in pore water and adsorbed loosely to surfaces, BD = redox-sensitive P mainly bound to oxidized Fe and Mn compounds, NaOH = P exchangeable against OH- ions or bound in organic matter, HCl = calcium phosphate minerals, and the residual is refractory organic P and nonextractable mineral P.

Sediment P was primarily bound to oxidized Fe/Mn compounds (BD fraction) and Ca phosphate minerals or acid-soluble organic P (HCl fraction; Fig 4). On average, 49.1% ± 1.8% of TP was associated with the BD fraction (range: 41–61%; *n* = 10) and 38.6% ± 2.1% with the HCl fraction (range: 25–47%). Given low Mn concentrations in Utah Lake sediment (≤0.06 wt. %; S2 Table), the BD fraction likely represents P associated with Fe rather than Mn. Likewise, given that the sediments are calcite-rich, the HCl fraction is likely dominated by P from calcium phosphate minerals rather than organic P. The other three fractions, including NH_4_Cl (loosely bound), NaOH (exchangeable P and P in organic matter), and the residual leach step (refractory organic P), accounted for an average of only 12.4% of TP in the sediment samples. Although concentrations were higher in the east samples, the percentage of P in each fraction was similar between the east and west samples.

### Water column and pore water phosphorus concentrations

**Fig 5:**
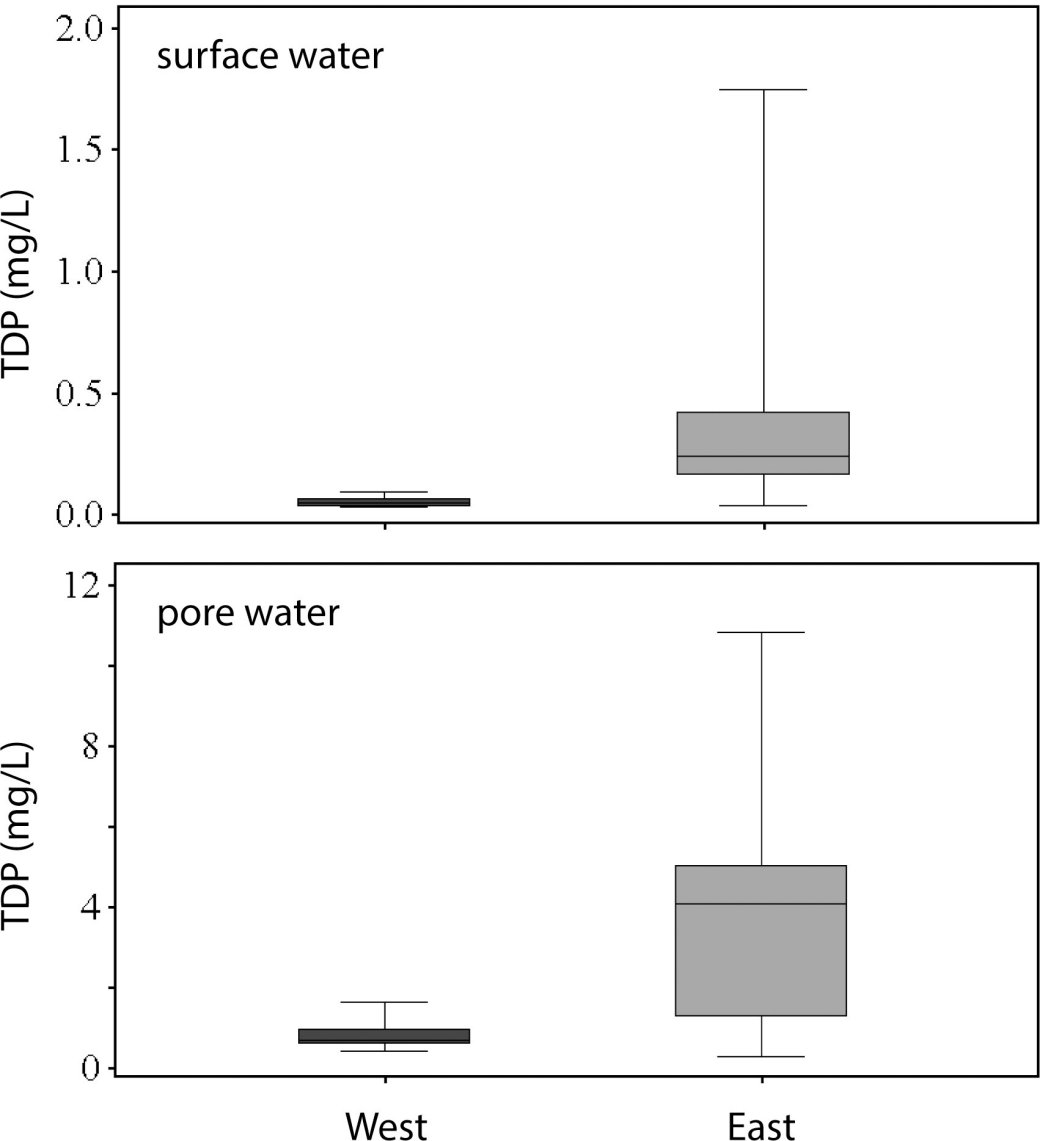
Total dissolved phosphorus (TDP) concentrations in surface water (top) and pore water (bottom). West samples (*n =* 13) were collected from sites 2–3, 5–10, and 14. East samples (*n =* 13) were collected from sites 1, 4, 11–13, and 15. For each sample type, TDP concentrations were averaged from the four sampling periods.

TDP concentrations in the water column and pore water were higher on the east side of Utah Lake near WWTP inputs relative to the west side (Fig 5). Concentrations in the east and west samples were significantly different for water column (t-test, *t* = 2.66, *p* = 0.021, df = 12.0) and pore water samples (t-test, *t* = 3.65, *p* = 0.003, df = 12.4). In the water column, TDP concentrations ranged from 0.04 to 1.74 mg/L in the east samples (0.43 ± 0.14 mg/L) compared with 0.03 to 0.09 mg/L in the west (0.05 ± 0.01 mg/L). Pore water TDP concentrations were approximately an order of magnitude higher than the water column concentrations and were higher on the east side relative to the west, ranging from 0.30 to 10.8 mg/L in the east samples (3.96 ± 0.85 mg/L) compared with 0.40 to 1.61 mg/L in the west (0.83 ± 0.11 mg/L).

### HABs prominent on east side of the lake

**Fig 6:**
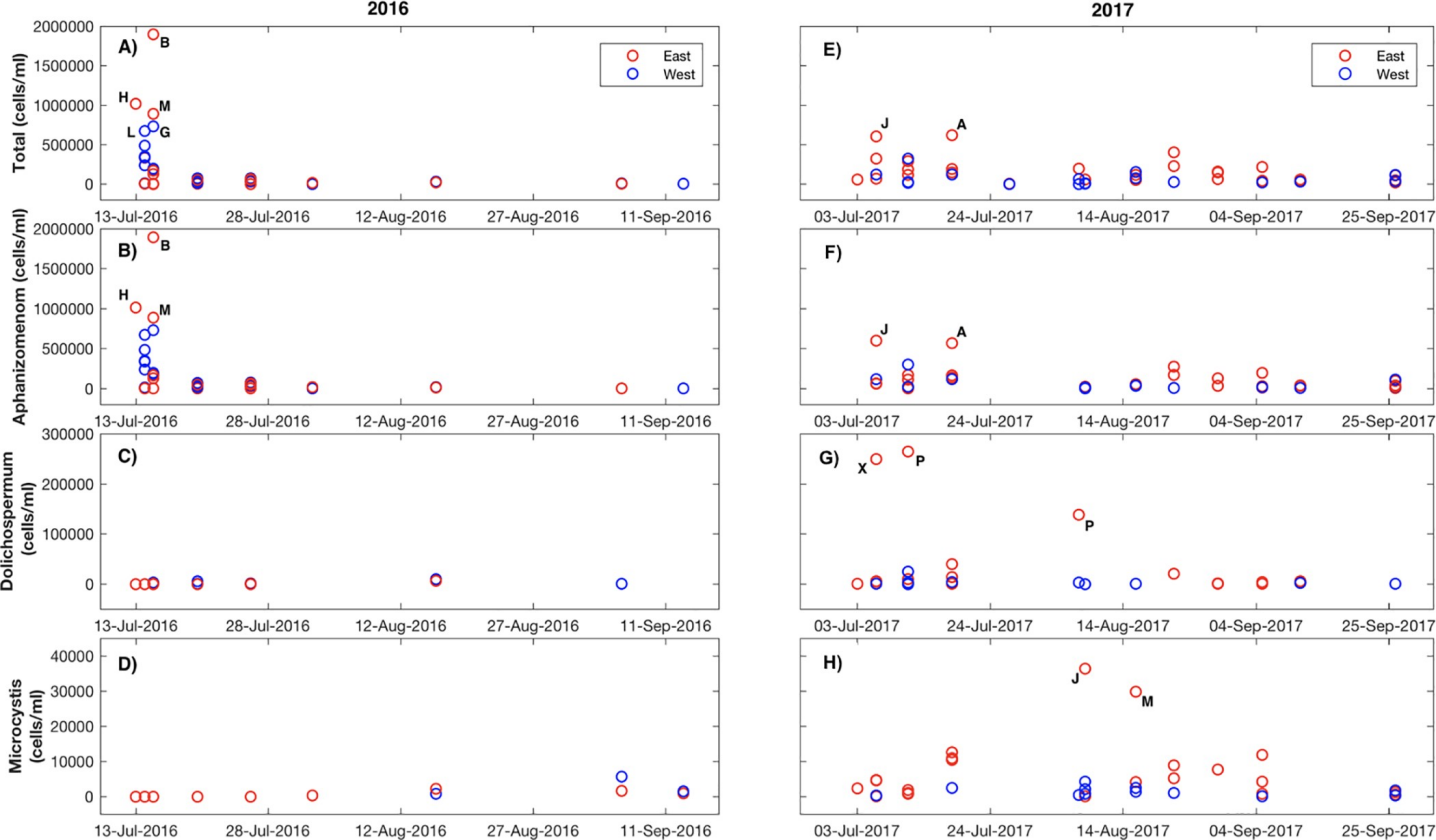
The abundance of total cyanobacteria in Utah Lake during summer 2016 and 2017. (A, E), *Aphanizomenon flos*-*aquae* (B, F), *Dolichospermum flosaquae* species (C, G), and *Microcystis aeruginosa* (D, H) cell counts for the summer of 2016 (*n =* 46) and 2017 (*n =* 52) across Utah Lake. Red circles and blue circles represent cell counts via semi-automated imaging flow cytometry from individual samples from the east and west side of the lake. Site labels correspond to locations shown in S2 Fig.

HABs were prominent on the east side of Utah Lake (Fig 6) where P concentrations were elevated in the sediment, pore water, and water column. A map showing sample site information is provided in the Supporting Information (S2 Fig). In the summer months of 2017, total cyanobacteria cell counts were at least 2.4-times higher in the east (143,244 ± 27,128) than west side (60,638 ± 18,155) of the lake (Fig 6E). Two cyanobacteria species, *Aphanizomenom flos*-*aquae* and *Dolichospermum flosaquae* dominated the total cell counts, with additional minor contributions of *Microcystis aeruginosa*. *Aphanizomenom flos*-*aquae* cell counts were consistently 3.2-times higher in the east (112,826 ± 25,270) relative to the west side (57,703 ± 17,117; Fig 6F), and along with *Dolichospermum flosaquae* contributed to blooms in July and August (Fig 6G). Alternatively, in early July of 2016, one immense bloom, predominantly of *Aphanizomenon flos*-*aquae*, occurred in three of the six east side locations where the cell counts were in the millions (34,193,162; 1,892,112; 1,418,070; Fig 6B). A bloom of *Microcystis aeruginosa* occurred along the east side of the lake later in the summer of 2017 in August (cell counts east = 7,211 ± 2,590; cell counts west = 910 ± 273). Blooms occurred infrequently in 2017 in west locations, but in 2016 one west side location experienced a minor bloom of *Microcystis aeruginosa* (5,631 cells) in late summer (Fig 6D).

## Discussion

### Spatial variability of phosphorus in Utah Lake

Phosphorus concentrations in pore water, water column, and sediment were generally highest on the east side of Utah Lake, reflecting legacy and current nutrient inputs from the urban area and WWTPs (Figs 1 and 5). Lake sediments contain legacy P from decades of nutrient inputs from the urban area, including WWTP effluent on the east side of Utah Lake and Provo Bay. Sediment TP concentrations in Utah Lake are similar to values reported for shallow, hypereutrophic Lake Taihu in China (range: 304–1553 mg/kg, *n* = 30) [30]. Elevated TP concentrations in pore water, which reflect elevated sediment TP concentrations, may contribute to P flux to the water column through diffusion or sediment resuspension. Notably, not all sediment on the east side of the lake contained elevated P concentrations. Sediment near the mouths of the Provo and Spanish Fork Rivers, which also coincide with WWTP outfalls (Fig 1), contained relatively low TP concentrations. The rivers likely contribute abundant silicate minerals, resulting in sediment with low capacity for P sorption (as indicated by the negative relationship between P and SiO_2_; Fig 2), or the rivers have low P concentrations that dilute the P budget. These are also areas where groundwater with low P concentrations discharges to the lakebed [21].

Total P concentrations in sediments were a function of proximity to WWTP outfalls and the mineral content or sorption capacity of the sediments. Sediment mineralogy was dominated by quartz, dolomite, and calcite (S2 Table), varying in abundance based on the relative inputs of detrital quartz and dolomite from tributaries and endogenic calcite that precipitates from the calcite-saturated water column. Organic matter, Ca, and Fe content control the amount of P retained in sediments through sorption or mineral precipitation (Fig 2). The role of organic matter in complexation of P is not clear since sequential leaching results showed a minimal amount of P in the organic matter (1M NaOH) fraction. The correlation may reflect co-located organic matter- and Ca- or Fe-rich sediments rather than interactions with organic matter. It is also possible that a fraction of the organic matter was coprecipitated with Fe(III) oxides and oxyhydroxides such that P in organic matter was released with the BD fraction rather than the 1 M NaOH fraction [31]. Notably, although Fe concentrations represented only a small fraction of total sediment composition (with Fe_2_O_3_ typically < 3% by weight; Fig 2), the sequential extraction results indicate that Fe retains approximately half of the sediment P by sorption to Fe (oxy)-hydroxides (Fe(OOH) ≈ P) (Fig 4). Sorption of P by Fe-oxide minerals was further demonstrated by an SEM image of sediment from Provo Bay, which shows the association of P with Fe-rich sediment grains (Fig 3). In Fig 3, the colored dot maps indicate the presence of specific elements (blue for Ca, green for Fe, and yellow for P) where P is dispersed across Ca- and Fe-rich grains.

The association of P with different minerals affects the subsequent mobility in sediments and potential flux to the water column. Phosphorus was primarily associated with Fe (Fe(OOH)) and Ca (CaCO₃ or Ca_10_(PO_4_)_6_(OH,F,Cl)_2_ ≈ P) from the BD and HCl fractions (Fig 4). These minerals act as sinks to sequester P from water column to the sediment [32, 33]. Co-precipitation of P with calcite and apatite minerals is strong at neutral to alkaline pH [34]. Utah Lake is an alkaline lake with pH values typically over 8 and is buffered by high bicarbonate concentrations. The Ca-associated P is likely precipitated with calcite (CaCO₃≈ P) or apatite (Ca_10_(PO_4_)_6_(OH,F,Cl)_2_) minerals. Although sediment chemistry is not kinetically conducive to precipitation of apatite, authigenic apatite mineral precipitation may occur as diatoms store polyphosphates inside their cells and form Ca phosphate minerals [35]. The majority of Ca-associated P may act as a permanent sink for P in Utah Lake with alkaline pH values. In contrast, the Fe-bound P pool is mobile as P may be released from sediments under anoxic conditions with the reductive dissolution of Fe-oxide minerals [36]. Anoxic conditions may develop locally near the sediment-water interface, particularly during summer when microbial activity is high. Although we did not evaluate seasonal differences in P fractionation in this study, it is possible that the Fe-bound fraction may decrease as P is released under summertime anoxic conditions. While flux rates from the sediment to overlying water column are unknown, the sediment-water interface is potentially a major controlling factor of P cycling in Utah Lake. Thus, quantifying P fluxes from sediment to overlying water is an important area for future study.

Elevated TDP concentrations in the water column and pore water were found on the east side of the lake, which also contained the highest TP concentrations in sediment. The relationship suggests that P flux from legacy sediments controls P concentrations in the pore water and water column or it reflects P movement from water to sediment. The sediments may act as a net sink or source depending on equilibrium P concentrations and changing geochemical conditions at the sediment-water interface. Notably, while sediment and water P concentrations were spatially related, there was less variability in sediment compared with water. Sediment concentrations varied six-fold across the lake (280–1710 mg/kg) whereas water column and pore water concentrations varied by nearly two orders of magnitude (0.07–1.9 and 0.3–10.8 mg/L, respectively). The greater variability in water concentrations may reflect biogeochemical processing in the water column and pore water. TDP concentrations were typically 2- to 4-times higher in pore water relative to the water column, likely because of reducing conditions in the sediment as P is released with reduced Fe [37].

### Phosphorus may lay a foundation for HABs

The elevated levels of P in the east side of Utah Lake appear to lay a foundation primarily for two species, *Aphanizomenon flos*-*aquae* and *Dilichosperma flosaquae*, to contribute to HABs. *Aphanizomenon* and *Dilichosperma* species are often triggered by TDP in the water column of otherwise P-limited systems [38, 39]. Both species may utilize high levels of P to generate biomass and satisfy their stoichiometric requirements for growth by fixing atmospheric N. *Aphanizomenon flos-aquae*, dominate HABs when nitrate:soluble reactive P ratios are relatively low [19, 40]. Little is known regarding the conditions that promote *Dolichospermum flosaquae* growth. *Dolichospermum* species may co-dominate blooms with other *Aphanizomenon* species like *Aphanizomenon gracile* and may be triggered by similar lake chemical conditions (Wood et al. 2017). TDP and TP are not the only factors leading to blooms as seen in the difference in bloom characteristics from 2016 to 2017. Multiple factors are necessary for blooms to occur, including N concentrations and meteorological conditions, but it seems that legacy P generated from multiple WWTPs over decades plays a role. Future research needs to identify the meteorological conditions and chemical thresholds, including those associated with multiple forms of P, allowing cyanobacteria species like *Aphanizomenon flos*-*aquae* to bloom in Utah Lake.

### Implications for lake restoration

Improvement in Utah Lake water quality would likely be delayed after a reduction in external P loads from WWTPs due to P release from lake sediments. Internal P cycling is commonly responsible for delayed response to decreasing external nutrient loads, with lakes typically reaching a new equilibrium within 10–15 years after nutrient reductions [6]. However, with high alkalinity, high pH, oxygenated and calcite-saturated waters, Utah Lake may represent the best-case scenario for a self-cleaning system to remove P from the water column by mineral precipitation in calcite. Ultimately the P-rich calcite may convert to thermodynamically stable apatite minerals and be further removed from the P cycle.

In addition to limiting external nutrient inputs, geo-engineering techniques could be considered for selected parts of Utah Lake to limit internal P cycling and HABs [41]. For example, a number of studies have shown field-scale success using lanthanum modified bentonite to limit P release from sediments [42]. Aluminum salts are another option for treating sediments but would likely not be effective in Utah Lake given the high pH and susceptibility of sediment resuspension [43, 44]. After carefully considering these and other factors, portions of the lake with elevated P concentrations in sediment, such as Provo Bay, could be targeted for P treatment. The combination of stepwise nutrient reductions and geo-engineering methods may be implemented as part of a cost-effective lake restoration program.

## Conclusion

Understanding the fate and mobility of phosphorus (P) in shallow lake sediments is essential for evaluating P cycling and potential impacts on water quality. In this study, we analyzed the water column, pore water, and sediment in Utah Lake to determine primary P sinks and the potential for internal P cycling. Our results suggest that majority of P in sediments is associated with calcite or apatite and Fe oxy/hydroxide minerals. Whereas the calcite- and apatite-bound P fraction is likely immobile in the sediments, the Fe-associated fraction is potentially released from sediments under reducing conditions. Sediment, pore water, and water column had the highest P concentrations on the east side of the lake near urban inputs. Pore water concentrations were an order of magnitude higher than the water column, demonstrating the potential for high flux rates between the bottom sediments and overlying water. Blooms of harmful cyanobacteria are also prevalent in areas of elevated P concentrations, indicating a link between legacy P pools and HABs. Our study has implications for water quality restoration plans of an increasing number of eutrophic lakes near developing cities, where nutrient pollution is critical.

## Supporting information

S1 Fig Representative XRD pattern for Utah Lake sediment showing prominent quartz and calcite peaks. This pattern is for sample 3-D collected in November 2016. The height of the mineral peaks represents the abundance of minerals present in the sample.
(TIF)

S2 Fig Sampling sites for cyanobacteria cell count data shown in Fig 6. (TIF)

S1 Table Sediment total P concentrations measured after sample digestion. (XLSX)

S2 Table Sediment chemistry and mineralogy. (XLSX)

S3 Table Sediment sequential extraction data. (XLSX)

S4 Table Water column chemistry. (XLSX)

S5 Table Pore water chemistry. (XLSX)
