# Supplementary figures and images for: Sediment potentially controls in-lake phosphorus cycling and harmful cyanobacteria in shallow, eutrophic Utah Lake
Source: PLoS One. 2019 Feb 14;14(2):e0212238. doi: 10.1371/journal.pone.0212238 (PMC6375609; doi:10.1371/journal.pone.0212238)

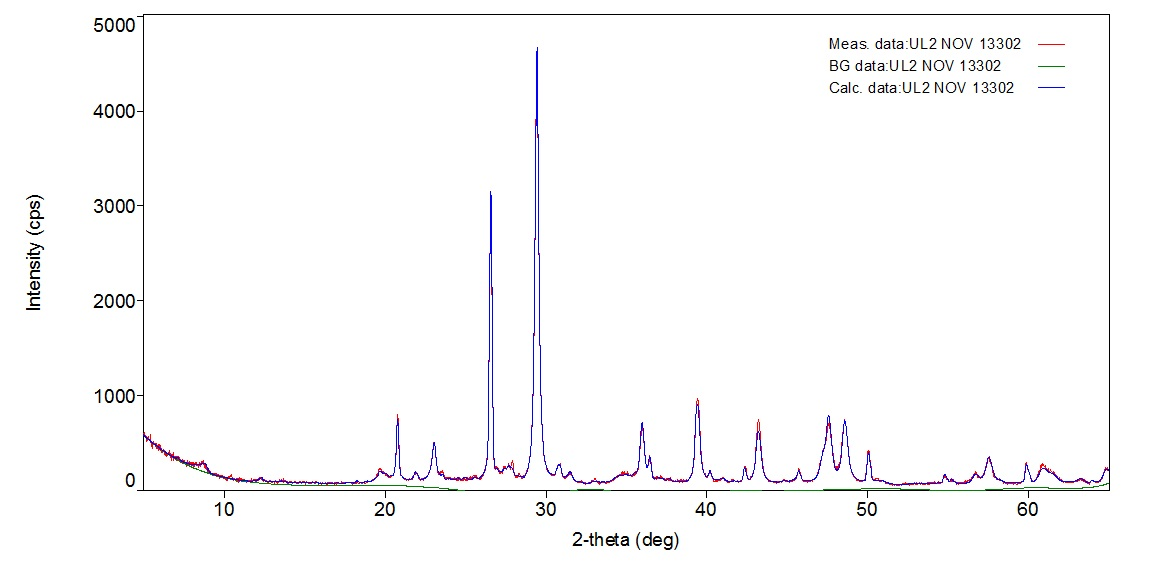

Supplement: S1 Fig — This pattern is for sample 3-D collected in November 2016. The height of the mineral peaks represents the abundance of minerals present in the sample. (TIF) [file pone.0212238.s001.tif]

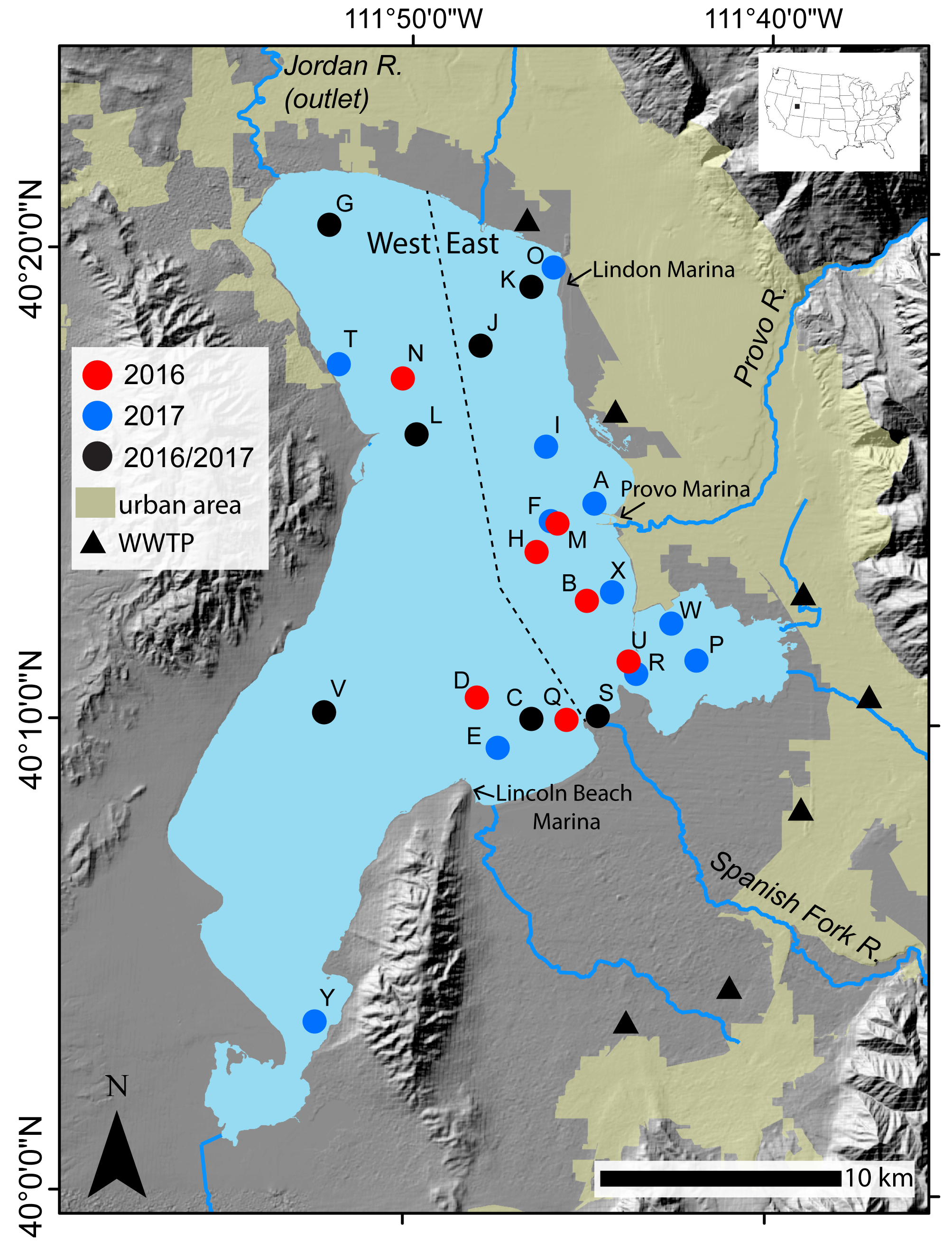

Supplement: S2 Fig — (TIF) [file pone.0212238.s002.tif]
